# Exocrine pancreatic insufficiency as an unusual extrarenal manifestation of proximal renal tubular acidosis associated with a novel *SLC4A4* mutation

**DOI:** 10.1007/s00467-025-06682-9

**Published:** 2025-01-27

**Authors:** Berfin Hasturk, Ayse Agbas, Ozlem Akgun-Dogan, Esra Karabag Yilmaz, Seha Saygili, Ömer Faruk Beşer, Nur Canpolat

**Affiliations:** 1 Department of Pediatrics, Istanbul University- Cerrahpasa, Cerrahpasa Faculty of Medicine, Istanbul, Turkey; 2 Department of Pediatric Nephrology, Istanbul University- Cerrahpasa, Cerrahpasa Faculty of Medicine, 34098 Istanbul, Turkey; 3 Department of Pediatric Genetics, Faculty of Medicine, Acibadem Mehmet Ali Aydinlar University, Istanbul, Turkey; 4 Rare Diseases and Orphan Drugs Application and Research Center (ACURARE), Acibadem Mehmet Ali Aydinlar University, Istanbul, Turkey; 5 Department of Pediatric Gastroenterology, Istanbul University- Cerrahpasa, Cerrahpasa Faculty of Medicine, Istanbul, Turkey

**Keywords:** Exocrine pancreas insufficiency, Steatorrhea, Fatty stool, Renal tubular acidosis, *SLC4A4*, NBCe1

## Abstract

Autosomal recessive proximal renal tubular acidosis (AR-pRTA) with ocular abnormalities is a rare syndrome caused by variants in the *SLC4A4* gene, which encodes Na/HCO3 cotransporter (NBCe1). The syndrome primarily affects the kidneys, but also causes extra-renal manifestations. Pancreatic type NBCe1 is located at the basolateral membrane of the pancreatic ductal cells and together with CFTR chloride channel, it is involved in bicarbonate secretion. In vitro models have demonstrated that mutations in the pancreatic type NBCe1 lead to a reduction in pancreatic bicarbonate secretion. Although elevated amylase levels have been observed in some cases, there is no evidence of symptomatic pancreas involvement in children with AR-pRTA. This report presents the case of a seven-year-old girl with AR-pRTA and exocrine pancreatic insufficiency. This novel presentation with a novel mutation in *SLC4A4* expands the extra-renal involvement in this rare disease. We recommend that these children be screened for exocrine pancreatic insufficiency.

## Case report

A three-year-old girl with renal tubular acidosis (RTA) was referred to our hospital for ongoing treatment challenges. The patient was born at 40 weeks with a birth weight of 2.5 kg. The prenatal history was unremarkable. The newborn screening test showed a positive immunoreactive trypsinogen result. The infant was fed with both breast milk and formula, and had diarrhea from birth. Fatty stools appeared after introduction of complementary feeding. A full evaluation was not conducted. At the age of 2.5 years, the patient presented with syncope and was diagnosed with distal RTA. Despite alkali treatment, severe acidosis resulted in multiple intensive care unit admissions. Family history revealed consanguineous parents with short stature, thoracic deformity, and blindness in the maternal grandfather and maternal aunt.

Upon admission to our center at age three, she was lethargic, exhibited significant growth retardation (height 79 cm, − 4.29 SD; weight 9 kg, − 3.68 SD) and neurodevelopmental delay, including the absence of independent sitting or walking. No evidence of rickets or hearing loss was observed. Lab tests showed hyperchloremic metabolic acidosis with a normal anion gap (pH 7.09, lactate 1 mmol/L, bicarbonate 8 mmol/L, sodium 136 mmol/L, chloride 115 mmol/L, potassium 4.5 mmol/L). Furthermore, urea, creatinine, and uric acid levels were normal, as were thyroid function tests. Urinalysis showed a specific gravity of 1010, pH 8, and no glucose. Additionally, the patient exhibited tubular proteinuria (0.9 mg/mg without albuminuria) and hypercalciuria (1.2 mg/mg). Kidney ultrasound confirmed bilateral mild nephrocalcinosis. The presence of tubular proteinuria and the need for high doses of alkali treatment were insufficient to confirm dRTA, and a genetic test was ordered.

The evaluation for fatty diarrhea revealed a positive steatocrit test, normal elastase (214 and 274 μg/g), normal pH (5.5), normal stool alpha-1 antitrypsin (0.7 mg/g), and negative stool reductase. Additionally, serum amylase (520 U/L) and lipase (617 U/L) levels were elevated, and the patient exhibited hypocholesterolemia (LDL 48 mg/dL, HDL 26 mg/dL, TG was normal) and increased pancreatic echogenicity on ultrasonography. These findings suggested exocrine pancreatic insufficiency. The initial differential diagnosis was cystic fibrosis (CF); however, there was no evidence of lung involvement, and sweat tests yielded normal results (15 mmol/L chloride). Given the hypocholesterolemia, endoscopy ruled out the possibility of abetalipoproteinemia or hypobetalipoproteinemia. She was treated with Creon (a pancreatic enzyme replacement therapy), lipid-soluble vitamins, and dietary modifications.

Ultimately, whole exome sequencing (encompassing copy number variations and single nucleotide variants) identified a novel, homozygous, nonsense, pathogenic variant, *SLC4A4* (NM_001098484.3), c.2024C > G, p.(Ser675*), which results in the replacement of serine at position 675 with a stop codon, consequently leading to the premature termination of the protein. In silico prediction tools, including DANN and MutationTaster, have classified this variant as deleterious. This variant has not been observed in population databases, such as gnomAD, ESP, or 1000 Genomes, and has not been reported in the medical literature. In accordance with the ACGS and ClinGen guidelines, this variant is classified as pathogenic, meeting the PVS1 and PM2 criteria, thereby confirming the diagnosis of autosomal recessive proximal renal tubular acidosis (AR-pRTA) with ocular abnormality syndrome. Furthermore, to investigate potential variants associated with exocrine pancreatic insufficiency, the Human Phenotype Ontology (HPO) term “Pancreatic insufficiency” (HP:0001738) was employed to generate a list of associated morbid OMIM genes using the HPO tool (https://hpo.jax.org/). Whole exome sequencing (WES) data were analyzed to assess all coding regions of the aforementioned genes, including both single nucleotide variants and copy number variations in *CFTR*, *SBDS*, *EFL1*, *eIF6*, *DNAJC21*, *SRP54*, *UBR1*, *COX4I2*, *PDX1*, *PTF1A*, *GATA6*, and *PNLIP*. This analysis evaluated all *CFTR* variants, ultimately excluding a diagnosis of CF.

Upon admission, the patient was taking potassium citrate but experienced gastrointestinal intolerance. Sodium bicarbonate was administered at a dose of 10–15 mmol/kg/day. During follow-up, bicarbonate levels remained between 15 and 20 mmol/L with a pH ranging from 7.26 to 7.32. Management of acidosis is still challenging. Higher doses led to elevated urea, creatinine, potassium, and phosphorus levels. By seven years of age, the estimated glomerular filtration rate was 90 mL/min/1.73 m^2^. Hypercalciuria had resolved and only a few punctate echogenicities were observed in the renal medulla. Since pancreatic enzyme supplementation, the patient experienced fewer fatty stools. There was a partial improvement in both weight (− 2.20 SD) and height (− 2.67 SD). Subsequently, enamel hypomaturation and band keratopathy were identified.

## Discussion

**Fig. 1:**
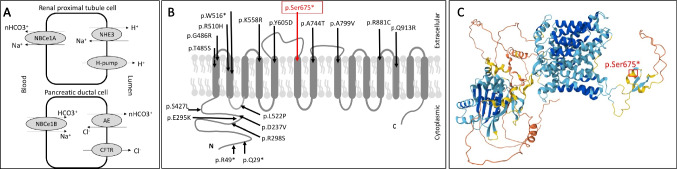
NBCe plays a role in bicarbonate absorption in renal proximal tubule cells whereas it also plays role in bicarbonate secretion with cystic fibrosis transmembrane regulatory channel in pancreatic ductal cells (**A**). Reported studies have found 17 nonsense/missense variants. Red arrow (the box) demonstrates the mutation in our case (**B**). 3D modeling of SLC4A4 protein and localization of variant. AlphaFold Protein Structure database (https://alphafold.ebi.ac.uk/entry/Q9Y6R1) was used to illustrate the location of the variant (**C**)

The *SLC4A4* gene, located in the 4q13.3, encodes the electrogenic sodium bicarbonate cotransporter type 1 (NBCe1) protein. The protein regulates bicarbonate secretion, absorption, and intracellular pH (Fig. 1). Three variants of the NBCe1 have been identified. The NBCe1-A variant is expressed in the basolateral membrane of the kidney proximal tubule, while the NBCe1-B variant is present in multiple tissues, including pancreas. The NBCe1-C variant is expressed in neurons and glia [1].

Mutations in the *SLC4A4* gene cause AR-pRTA with ocular abnormality syndrome in humans. A limited number of case reports have been published [1–3]. Correcting acidosis is almost impossible because substantial alkali treatment may lead to non-compliance, GIS intolerance or exceed the proximal tubular bicarbonate reabsorption capacity, leading to an increased urinary loss [3]. The most common extra-renal findings include growth retardation and ocular findings such as glaucoma, band keratopathy, or cataract. Other reported features include enamel hypoplasia, neuromotor developmental delay, neurological findings (epilepsy, paraplegia, migraine, ataxia), and basal ganglia calcification. Treating acidosis with alkalinization therapy has a limited effect on extrarenal findings.

Elevated serum amylase levels have been sporadically reported [2, 4]. However, there is no evidence of exocrine pancreatic insufficiency. Exocrine pancreas insufficiency is defined as the inadequate secretion of pancreatic enzymes and/or bicarbonate-rich fluid. The bicarbonate is essential for maintaining the optimal luminal pH, which is required for pancreatic enzyme activity. The best known genetic cause of exocrine pancreas insufficiency is CF. CF is characterized by impaired secretion of chloride and bicarbonate in the pancreatic ductal system. CFTR-mediated chloride secretion activates NBCe1-B activity through cAMP, which mediates bicarbonate uptake across the basolateral membrane of pancreatic duct cells [5]. In CF patients, impaired CFTR function leads to decreased electrogenic driving force, which reduces NBC-driven basolateral bicarbonate entry into the duct cells [5]. Therefore, it can be speculated that abnormalities in pancreatic NBCe1-B function may similarly reduce ductal bicarbonate secretion as seen in CF. Satoh et al. [6] have studied this hypothesis. They showed that NBCe1-B is the dominant variant in the human pancreas cells, abundantly expressed in the basolateral membrane of intercalated duct cells along the entire duct system. They also showed defective bicarbonate secretion in the human pancreas duct cells with R342S and R554H pancreatic-type mutations. They speculated that, due to the rarity of AR-pRTA cases, pancreatic phenotype may remain underrecognized or undetected.

In conclusion, a mutation in the *SLC4A4* gene that disrupts pancreatic NBCe1-B function may impair pancreatic ductal bicarbonate secretion, leading to a CF-like phenotype, as observed in our patient. This case highlights the importance of increasing clinician awareness of extra-renal manifestations, particularly pancreatic involvement, in this rare disorder. Given NBCe1’s broad tissue distribution, as demonstrated in vitro, clinicians should remain vigilant during follow-up for potential involvement of other tissues not yet well-documented, such as the heart and lungs.

## Summary

### What is new?

- Exocrine pancreas insufficiency, manifested as fatty stool, lipid malabsorption, and malabsorption of lipid-soluble vitamins, may be an extrarenal finding associated with AR-pRTA due to a novel *SLC4A4* mutation.

## Data Availability

Data can be shared upon a reasonable request from the corresponding author.
